# A case series report on successful management of patients with COVID-19-associated lymphopenia and potential application of PG2

**DOI:** 10.3389/fmed.2022.1009557

**Published:** 2022-11-04

**Authors:** Wei-Yao Wang, Yuan-Ti Lee, Yao-Tung Wang, Ji-Zhen Chen, Su-Yin Lee, Shih-Ming Tsao

**Affiliations:** ^1^Division of Infectious Disease, School of Medicine, Chung Shan Medical University Hospital, Chung Shan Medical University, Taichung, Taiwan; ^2^Division of Chest Medicine, School of Medicine, Chung Shan Medical University Hospital, Chung Shan Medical University, Taichung, Taiwan; ^3^Department of Internal Medicine, Chung Shan Medical University Hospital, Taichung, Taiwan; ^4^Infection Control Center, Chung Shan Medical University Hospital, Taichung, Taiwan

**Keywords:** COVID-19, lymphopenia, *Astragalus* polysaccharides, PG2, neutrophil-to-lymphocyte ratio

## Abstract

**Background:**

Lymphopenia and the resultant high neutrophil-to-lymphocyte ratio (NLR) are hallmark signs of severe COVID-19, and effective treatment remains unavailable. We retrospectively reviewed the outcomes of COVID-19 in a cohort of 26 patients admitted to Chung Shan Medical University Hospital (Taichung City, Taiwan). Twenty-five of the 26 patients recovered, including 9 patients with mild/moderate illness and 16 patients with severe/critical illness recovered. One patient died after refusing treatment.

**Case presentation:**

We report the cases of four patients with high NLRs and marked lymphopenia, despite receiving standard care. A novel injectable botanical drug, PG2, containing *Astragalus* polysaccharides, was administered to them as an immune modulator. The decrease in the NLR in these four patients was faster than that of other patients in the cohort (0.80 vs. 0.34 per day).

**Conclusion:**

All patients recovered from severe COVID-19 showed decreased NLR and normalized lymphocyte counts before discharge. Administration of PG2 may be of benefit to patients with moderate to severe COVID-19 and lymphopenia.

## Introduction

Lymphopenia accompanying inflammatory cytokine storms is frequently reported (35–83%) in patients who with SARS-CoV-2 infection (1–4). A decreased absolute lymphocyte count (ALC) and an increased neutrophil-to-lymphocyte ratio (NLR) is indicative of severe COVID-19 and are associated with a poor prognosis (5, 6). There is no medical treatment currently available to reverse lymphopenia and prevent disease progression (7). In this context, we report our experience of the effectiveness of PG2 in the management of such patients; PG2^®^ lyophilized injection is an approved botanical drug containing bioactive polysaccharides of dried *Astragalus membranaceus* (Chinese: Huang-Chi) roots and was originally used for treatment of cancer-related fatigue. According to the approved label information and previous publications (8), PG2 or *Astragalus* polysaccharides can elicit a broad spectrum of therapeutic effects, including modulation of the immune system. More specifically, PG2 has been shown to induce differentiation of splenic dendritic cells (DCs), expand the CD11c*^high^*CD45RB*^low^* DC pool, increase the Th1/Th2 ratio, and enhance the T cell-mediated immunity *in vitro* (9). Previous clinical studies suggest that PG2 also modulates the inflammatory cascade *via* suppression of pro-inflammatory cytokines (10).

The Taiwanese government has employed strict boarder control and no community-acquired cases of COVID-19 were observed until the COVID-19 epidemic in May 2021, in which a total of 16,278 confirmed cases were reported, with 851 deaths. A total of 26 patients (11 males and 16 females), with COVID-19, were admitted to Chung Shan Medical University Hospital (CSMUH) in central Taiwan between May 19, 2021 and June 10, 2021. Taiwan. Table 1 shows the demographic and clinical characteristics and the outcomes of these patients. Nine patients were categorized as mild or moderate severity, and the other 16 patients were categorized as severe or critical, according to the World Health Organization (WHO) criteria (11). We also evaluated the clinical severity, on admission and during the hospitalization period of the 26 patients, using a widely accepted 7-point ordinal scale (12). Patient with severe disease were older in age, with more comorbidities including diabetes, cardiovascular (CV) risk factors and histories of malignancies when compared to those with mild to moderate disease on admission. Only one of our 26 patients died after refusing treatment. The other 25 patients, including 9 with mild/moderate illness and 16 with severe/critical illness, recovered with adequate therapy. A cycle threshold (Ct) of SARS-CoV-2 RT-PCR greater than 30 indicated viral negative and/or non-contagious for discharge.

**TABLE 1:** Clinico-demographic data and outcome profiles of 26 patients with laboratory-confirmed COVID-19.

Characteristic of cases	Total cases (%) (26 cases, 100%)	Severity (%)		
		
		Mild to moderate (9 cases, 34.6%)	Severe without PG2 treatment (13 cases, 50.0%)	Severe with PG2 treatment (4 cases, 15.4%)
Age (years) (±SD)	49.8	30.7	59.5	54.5
Male sex	11 (42.3)	4 (44.4)	5 (38.5)	2 (50)
Comorbidities^a^	13 (50.0)	3 (33.3)	9 (69.2)	1 (25.0)
Diabetes mellitus	4 (15.4)	0 (0)	4 (30.8)	0 (0)
CV risk factor	8 (30.8)	1 (11.1)	6 (46.2)	1 (25.0)
Malignancy	3 (11.5)	0 (0)	3 (23.0)^b^	0 (0)
Day from illness onset to dyspnea (±SD)	5.5 ± 3.1	3.9 ± 2.8	6.2 ± 2.6	7.0 ± 4.5
Systolic blood pressure on admission, mmHg (±SD)	131.1 ± 16.6	123.6 ± 15.9	134.1 ± 15.1	138.3 ± 20.8
Respiratory rate > 24 breaths/min on admission	7 (26.9)	0 (0)	5 (38.5)	2 (50)
Disease severity on admission (1–7 points) (±SD)	4.7 ± 1.5	2.9 ± 0.6	5.8 ± 0.4	5.3 ± 1.5
Highest disease severity (1–7 points) (±SD)	4.8 ± 1.5	2.9 ± 0.6	5.8 ± 0.4	5.8 ± 0.5
Oxygen therapy on admission	12 (46.2)	0 (0)	9 (69.2)	3 (75.0)
Mechanic ventilation	5 (19.2)	0 (0)	4 (30.8)	1 (25.0)
Death	1 (3.8)^c^	0 (0)	1 (7.7)	0 (0)

^a^No patient with comorbidity of chronic obstructive pulmonary disease (COPD), chronic kidney disease (CKD), chronic liver disease (CLD), and current smoking.

^b^Endometrial carcinoma, breast cancer, and prostatic cancer for each one patient.

^c^The patient died after refusing treatment and issuing a “do not resuscitate” order.

Four patients in this cohort had marked lymphopenia, despite standard care. We treated them with PG2 injections, after receiving emergency authorization from the hospital’s institutional review board. Written informed consent was obtained from the patients or their legal guardian, prior to initiation of this treatment. The dosage of PG2 was as follows: 500 mg dissolved in 500 mL normal saline *via* intravenous infusion for 2.5–3.5 h, once every other day (QOD). Summaries of the clinical characteristics, outcomes and laboratory and radiologic findings are shown in Table 2 and Supplementary Table 1, respectively.

**TABLE 2:** Clinico-demographic data, treatment modalities, and outcomes of four patients with severe/critical COVID-19 infection who received PG2 treatment.

Characteristic	Case I	Case II	Case III	Case IV
Age (years)	46	50	47	75
Sex	F	M	M	F
Comorbidities	None	Hypertension, hyperlipidemia, stroke	None	None
Days from onset of illness to dyspnea	4	7	4	8
Systolic pressure on admission (mmHg)	122	126	137	168
Respiratory rate > 24 breaths/min	No	Yes	No	Yes
SpO_2_ (%), room air	88	90	96	82
Disease severity on admission (1–7 points)	6	6	3	6
Highest disease severity during hospitalization (1–7 points)	6	6	5	6
Antibiotic treatment	Yes	Yes	Yes	Yes
Corticosteroid treatment	Yes	Yes	Yes	Yes
Remdesivir treatment	Yes	Yes	Yes	Yes
Tocilizumab treatment	Yes	Yes	No	Yes
Oxygen therapy on admission	HFNC (15 L/min)	HFNC (15 L/min)	O_2_ nasal cannula (3 L/min)	No
Mechanic ventilation	No	No	No	Yes
Lymphopenia prior to PG2 treatment ^※^	Gr. 2	Gr. 3	Gr. 2	Gr. 3
Outcome	Recovered	Recovered	Recovered	Recovered

^※^Grading according to CTCAE v5.0 with the adverse event term “lymphocyte count decreased.”

## Case descriptions

### Case I

A 46-year-old woman without comorbidities, was transferred from a government quarantine facility to our hospital; she had marked oxygen desaturation on admission. Dexamethasone 6 mg once per day (QD) was given for 7 days on admission. On the second day, a standard 5-day course of remdesivir (200 mg on day 1, followed by 100 mg from days 2 to 5) was initiated; in addition, a single dose of tocilizumab 8 mg/kg was also administered to manage the patient’s high C-reactive protein (CRP) level (9.6 mg/dL), in accordance with the standard guidelines of the Taiwan Center for Disease Control (Taiwan CDC) (13). However, on day 3, laboratory data showed a higher NLR (4.9) than on admission and the patient’s Chest X-ray (CXR) showed persistent bilateral infiltrates. She was treated with a PG2 infusion (500 mg in 500 mL 0.9% saline) QOD for five doses. No adverse reactions were noted. Her clinical condition improved and a CXR also showed improvement. Laboratory tests showed an increase in the lymphocyte count, and lactate dehydrogenase (LDH) and CRP levels within the normal range. The patient’s NLR decreased to 1.9 on day 5 of hospitalization and remained stable (1.3–2.2) below the cut-off value of 3.5 throughout the remaining hospitalization period. She was discharged 10 days after admission with a Ct value: 38 for SARS-CoV-2 PCR.

### Case II

**FIGURE 1:**
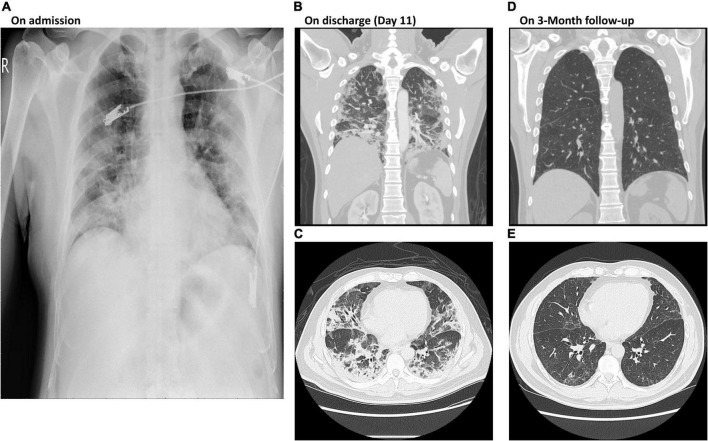
Chest X-rays of Case II on admission and at 3-month follow-up post discharge. **(A)** Diffuse bilateral lung infiltration shown by chest X-ray on admission. **(B,C)** Patchy shadows of high density in lobule were detected by CT images on discharge at day 11. **(D,E)** Clear CT images show both lobules without patchy shadows at 3-month follow up post discharge. CT, computer tomography; PCR, polymerase chain reaction.

A 50-year-old man was admitted to the respiratory intensive care unit (ICU) on account of dyspnea, hypoxia (SpO_2_ 90% on oxygen 3 L/min *via* nasal cannula) and fever (>38°C). He was administered standard therapy including remdesivir (200 mg on day 1, followed by 100 mg from day 2 to day 5), dexamethasone 8 mg QD, and a single dose of tocilizumab 640 mg. However, his clinical condition deteriorated with progressive dyspnea and hypoxia on day 3. The oxygen treatment was switched to high flow nasal cannula (HFNC) with a positive end-expiratory pressure (PEEP) of 8 cm H_2_O. PG2 500 mg QOD was then initiated since day 4. His condition improved and the oxygen support was switched from a HFNC to a nasal cannula on day 5. He was discharged on day 11 after his condition stabilized and his polymerase chain reaction (PCR) test showed a negative result. On admission, his NLR and ALC were 10.5 and 381 cells/μL, respectively. On day 9 after administration of PG2, his NLR decreased to 5.26 and his ALC increased to 1,608 cells/μL. On day 13, PCR showed negative result of viral RNA. A total of 6 vials of PG2 was given in 10 days and the patient was discharged after 18 days of hospitalization. He had follow-up chest computed tomography (CT) scans 1 and 3 months after discharge. The CT revealed lung consolidation and gradual resolution of the fibrosis, without any residual lesion (Figure 1).

### Case III

A 47-year-old man, without comorbidities, was transferred from a government quarantine facility to our hospital. Initially, his O_2_ saturation remained above 94%, after insertion of a O_2_ nasal cannula. However, his NLR increased to 5.8 and his ALC decreased to 611 cells/μL. His CRP level increased from 2.951 to 6.421 mg/dL 10 days post admission, despite the administration of standard therapy (remdesivir, corticosteroids, and antibiotics). PG2 treatment (500 mg QOD) was initiated on day 10 of hospitalization. His NLR decreased to 4.1 and his ALC increased to 1,118 cells/μL 3 days after starting PG2 treatment. His laboratory test results showed a decrease in LDH and CRP levels. On day 11, his Ct value was 35 for SARS-CoV-2 PCR. The patient was discharged from the hospital on day 17.

### Case IV

A 75-year-old woman, without comorbidities, was transferred to our hospital 8 days after the onset of COVID-19 symptoms, which included intermittent fever. Her oxygen saturation decreased progressively while receiving oxygen *via* a non-rebreathing mask and she showed clinical signs of respiratory distress, so she was intubated and admitted to the ICU. A standard course of remdesivir (200 mg on day 1, followed by 100 mg from day 2 to day 5), and one dose of tocilizumab 8 mg/kg were administered. Within the first 4 weeks of hospitalization, she contracted multiple nosocomial and secondary infections, including fungal infection, herpes zoster, and a urinary tract infection. Furthermore, she developed acute kidney failure and hemodialysis was initiated. Repeated attempts were made to wean her from the ventilator and regular hemodialysis was continued. On day 30 of hospitalization, laboratory tests showed a high NLR (40.65) and an ALC of 303 cells/μL; CXR revealed increasing infiltrations bilaterally in addition to a left-sided pleural effusion. She was administered a PG2 infusion 500 mg QOD for five doses until her clinical condition stabilized. Her CXR improved after the PG2 treatment. Prior to initiation of PG2 therapy, her NLR was 11.8 on admission and increased progressively, reaching 50 on day 24. Her NLR did not improve after administration of antibiotics and steroid treatment. After completion of the PG2 therapy, laboratory tests showed improvement in the NLR (21.98) and ALC (576 cells/μL). Her SARS-CoV-2 PCR result showed Ct: 28 on day 15 and Ct: 38 on day 38. She was hospitalized for a further 2.5 months due to continuous lung infiltration. She experienced recurrent infections but her clinical condition gradually improved and she was weaned from the ventilator and hemodialysis was discontinued prior to discharge. She was discharged from the hospital after 132 days of admission, and was provided with home care with low flow supplemental oxygen *via* a nasal cannula.

## Discussion

**FIGURE 2:**
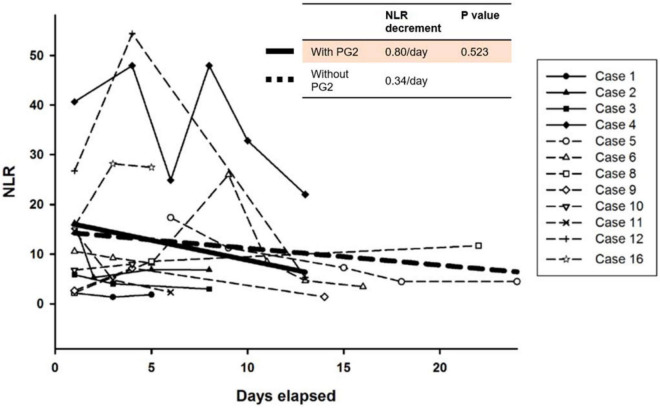
Time course changes of neutrophil-to-lymphocyte (NLR) ratio in patients with severe COVID-19. The bold straight line indicates estimates of the NLR decrease per day by linear mixed model in patients treated with PG2, whereas the dotted straight line indicates integrated trend in the NLR in patients who were not treated with PG2.

The 25 patients admitted to with COVID-19 received standard care guided by the protocol adopted from guidelines of Taiwan CDC including the use of supplemental oxygen, prone positioning, and systemic sequential treatment with corticosteroids, remdesivir, and tocilizumab, as well as anticoagulation agents as needed (13). However, there is no specific clinical guideline for patients with persistent respiratory distress, lymphopenia, and a high NLR. Among the 25 patients, 16 were considered to have severe disease based on an SpO_2_ of <93% breathing room air and requiring high flow supplemental oxygen or ventilator support. Twelve of the 16 patients with severe disease had an ALC < 1,000 cells/μL during their hospitalization. Four patients received PG2 in addition to standard care, and 8 patients who received only standard care served as control group for comparison. Four patients did not experience a decrease in their ALC to <1,000 cells/μL. The NLR of these patients remained relatively low and their clinical condition did not deteriorate during their hospitalization. Patients who received PG2 showed faster decrease in the NLR than those who did not receive PG2. A linear mixed model was used to compare the tendency of time course change in the NLR among patients with an ALC < 1,000 cells/μL. Patients who received PG2 had an average NLR decrease of 0.80 per day, compared to those who did not receive PG2 had an average NLR decrease of 0.34 per day overtime (Figure 2). Although the comparison was not statistically significant (*P* = 0.523), the tendency for ALC to increase and NLR to decrease after administration of PG2 warrants further investigation and clinical development on its immune modulatory effects.

There is a need for novel therapies for treating patients with COVID-19 and lymphopenia as currently there is no treatment available for treating lymphopenia and T-cell depletion in routine clinical practice (7, 14). A similar situation also arises in patients with other infectious diseases and during cancer therapy (15). A previous study revealed a similar pattern, and showed that patients with severe COVID had significantly higher neutrophil counts, lower lymphocyte counts, higher NLRs, and lower percentages of monocytes, eosinophils and basophils (16). In addition to a significant reduction in both CD4 + and CD8 + lymphocyte subsets has been observed in patients with severe COVID-19, including lower CD8 + lymphocyte count, and an increase in the CD8 + lymphocyte count was associated with clinical improvement (17). In a previous study, we demonstrated the immunomodulatory effect of PG2 for treating lung cancer patients with moderate to severe fatigue while receiving immune checkpoint inhibitors (18). Compared with patients without PG2, those who received PG2 were more likely to experience a lowering of their NLR to ≤ 3.5, which was probably attributable to the rapid increase in lymphocytes during their treatment. Furthermore, a previous study showed that *Astragalus* polysaccharides suppress pro-inflammatory cytokines, including interleukin 6 and tumor necrosis factor alpha (19), which have both been found to play a role in the cytokine storm of acute respiratory distress syndrome, a major cause of COVID-19-associated death. Further studies reveal that PG2 promoted the polarization of THP-1-derived macrophages into an anti-inflammatory phenotype, and inhibited LPS-induced cytokine release. This suggests that PG2 could potentially prevent viral infection of host cells and prevent the development of severe COVID-19 *via* regulation of macrophage activity, similar to previously reported effects with *Astragalus* polysaccharides (19). We believe that PG2 may also restore functionality to exhausted lymphocytes and restore immune homeostasis after severe COVID-19. However, randomized controlled trials are required to confirm the beneficial effects of PG2.

There are several limitations to the present study. Only four patients were treated with PG2 due to the limited number of patients with severe COVID-19 admitted to our hospital. The results were obtained retrospectively. The possibility of selection bias cannot be ruled out.

In conclusion, PG2 demonstrated potential benefit in promoting recovery of lymphocytes in patients with severe COVID-19 and COVID-19-associated lymphopenia. No drug related adverse reactions were observed. Randomized controlled trials are needed to investigate the role of PG2 in controlling lymphopenia in patients with COVID-19.

## Data availability statement

The original contributions presented in this study are included in the article/Supplementary material, further inquiries can be directed to the corresponding author.

## Author contributions

S-MT led the care of COVID-19 patients and obtained emergency authorization from the institutional review board for use of novel therapeutic agents. W-YW, Y-TL, and Y-TW took care of the patients. J-ZC and S-YL collected data. All authors reviewed, edited, and approved the final manuscript.
