# Supplementary material for: A case series report on successful management of patients with COVID-19-associated lymphopenia and potential application of PG2
Source: Front Med (Lausanne). 2022 Nov 4;9:1009557. doi: 10.3389/fmed.2022.1009557 (PMC9671954; doi:10.3389/fmed.2022.1009557)
Supplement: Supplementary file 1 [file Table_1.docx]

Supplementary Material

**Supplementary Table 1S. Initial Laboratory and radiologic findings of four patients with severe COVID-19 infection who received PG2 treatment**

| Laboratory/Case | Case I | Case II | Case III | Case IV |
| --- | --- | --- | --- | --- |
| WBC, cells/µL | 5540 | 4480 | 4990 | 12740 |
| Lymphocyte, cells/µL | 781 | 381 | 828 | 943 |
| Neutrophil/lymphocyte ratio (NLR) | 4.26 | 10.5 | 4.48 | 11.85 |
| Hb, g/L | 13.6 | 13.4 | 14.5 | 10.6 |
| Platelets counts, 10^9^/cells | 147 | 198 | 174 | 246 |
| D-dimer, mg/L | 505 | 531.09 | 246.73 | 2217.5 |
| Blood urea nitrogen (BUN), g/L | 5 | 15 | 9 | 10 |
| Creatinine (Cr), g/L | 0.5 | 1.07 | 1.01 | 0.57 |
| Aspartate transferase (AST), U/L | 67 | 39 | 31 | 48 |
| Alanine transferase (ALT), U/L | 31 | 37 | 41 | 27 |
| Lactate dehydrogenase, U/L | 350 | NA | NA | NA |
| Ferritin, ng/mL | NA | 654.8 | 484.1 | 571.3 |
| High sensitivity C-reactive protein (Hs-CRP), mg/L | 9.62 | 6.279 | 0.425 | 6.005 |
| Creatinine kinase, U/L | 48 | 84 | 246 | 86 |
| Bilateral involvement of chest radiographs | Yes | Yes | Yes | Yes |
| Cycle of threshold (Ct) of RT-PCR at admission for respiratory specimen | NA | <30 | 18 | NA |

NA, not available at admission, RT-PCR, reverse transcription polymerase chain reaction.
